# Displacing Problems: A Constructivist Grounded Theory of Problematic Pornography Use

**DOI:** 10.1007/s10508-025-03214-2

**Published:** 2025-09-11

**Authors:** James Binnie, Ian Albery, Paula Reavey

**Affiliations:** 1 Division of Psychology, School of Applied Sciences, London South Bank University, 103 Borough Road, London, SE1 0AA UK; 2 The South London and Maudsley NHS Foundation Trust, London, SE5 8AZ UK

**Keywords:** Problematic pornography use, Addiction, Constructivist grounded theory, Pornography

## Abstract

Research indicates that pornography is not inherently harmful for the individual; however, many users consider their use to be problematic. The majority of research concerning problematic pornography use (PPU), often referred to as pornography addiction, discusses nomenclature rather than having an applied focus. Given the lack of theoretical development in this area, a constructivist grounded theory was undertaken with the aim of creating an understanding of the development and maintenance of PPU. All participants were required to have self-reported PPU and were recruited from online sources. A total of 258 journals of pornography use and 21 semi-structured interviews were completed. Five interlinked categories were constructed from the data. All participants were seen as having distinct problems prior to their self-reported PPU. Over time, their pornography use changed function, from enjoyment to using habitually, instrumentally, or as a form of emotional regulation. The participants’ discovery of “having PPU” occurred by proxy, through an external means. When participants reached out for information, they were met with an addiction narrative and consequently saw themselves as addicted to pornography. Once this narrative was internalized, the participants displaced their distinct problems, the participants now saw their main problem as addiction, rather than their preexisting distinct problems. This process of displacement was conceptualized as the core category in this grounded theory in that it led participants to committing to a mission, attempting to conquer their addiction. Having once embarked upon their mission, the underlying distinct problems became secondary to the participants, often seen as resulting from their pornography use. The constructivist grounded theory was situated within current theory and research. Some aspects of the grounded theory were judged as having similarities to existing theories, but when taken as a whole it is proposed that the grounded theory, with its focus upon displacement, is original, having clear implications for future research and clinical application.

## Introduction

Lifetime prevalence of pornography consumption is 96% in men and 61% in women (Döring et al., 2017). The use of pornography has increased in line with developments in technology—in 2023 there were 125 million visits per day to Pornhub (Pornhub, 2023). Research on compulsive, problematic, or addictive pornography has increased exponentially in recent years (Grubbs et al., 2020). Although pornography is not inherently problematic, the research literature suggests that it can become problematic for certain people (Cooper et al., 2000; Ross et al., 2012; Twohig et al., 2009). PPU can be defined as any use of pornography that leads to significant negative interpersonal, intrapersonal, or extrapersonal consequences for the user (Sniewski et al., 2018). Ley et al. (2014) proposes the prevalence of PPU to be 0.58% and 0.43% of men and women, respectively.

Classifying PPU can be challenging as it is often considered a behavioral addiction (Marks, 1990), frequently subsumed under sex addiction (Orzack & Ross, 2000; Rosenberg et al., 2014), or internet addiction (Block, 2008; Brand et al., 2016). It can also be classed as an impulse control disorder/sexual impulsivity/sexual compulsivity/compulsive sexual behavior disorder (Cooper et al., 1999; Grant & Potenza, 2007; Kraus et al., 2018; Mick & Hollander, 2006). The majority of studies focusing upon PPU discuss such nomenclature (see Binnie & Reavey, 2020 for a review).

Intervention studies are well described within recent systematic reviews (Antons et al., 2022; Roza et al., 2023; Sniewski et al., 2018). The findings of these reviews suggest there is some limited evidence for treating PPU, with Paroxetine/Naltrexone considered the most effective medical approaches, and acceptance and commitment therapy the most effective psychological approach.

A popular, non-evidenced based intervention for PPU is “rebooting.” Rebooting originated on Reddit sites and has subsequently expanded onto social media platforms. Some of the larger rebooting communities are now commercial enterprises. In summary, rebooting supports the notion of refraining from using pornography and masturbation to overcome pornography addiction (see Chasioti & Binnie [2021] and Taylor & Jackson (2018), for a detailed examination of online reboot communities).

There are theoretical models of PPU in the academic literature. The moral incongruence model (Grubbs et al., 2019) proposes PPU occurs when an individual violates their moral belief system by using pornography. Moral incongruence has been found to associated with increased psychological distress and perceived addiction (Grubbs & Perry, 2019). The interaction of person-affect-cognition-execution (I-PACE) model (Brand et al., 2016) is a theoretical framework describing the psychological and neurobiological processes thought to be responsible for the addictive use of internet applications including pornography. Both models have merit but do not seem to fully encapsulate the processes involved in the etiology and persistence of PPU; they are either too specific or generic. However, recent research by Bőthe et al. (2024) identified several risk factors for the development of PPU; these include the frequency of using pornography, using pornography to cope with negative emotions, and the aforementioned aspect of moral incongruence. It can therefore be summarized that there are indications from existing research as to the development and maintenance of PPU, but no clear theoretical model.

The lack of a comprehensive model hinders psychological practitioners in their work with clients presenting with PPU. A psychological model allows for a wider conceptualization or formulation of the clients’ difficulties and gives a direction for the therapeutic work. The aim of this study was to develop an applied model of PPU. The research question was: “How do adults experience their self-reported problematic pornography use, and how can sense be made of the development and maintenance of their problems?”

## Method

A constructivist grounded theory (CGT) methodology was used throughout. Grounded theory is a systematic method of qualitative research with the primary aim of generating new theory to explain phenomena (Strauss & Corbin, 1990). CGT (Charmaz, 2006, 2014) posits theories are not discovered; they are ultimately constructed by the researcher. CGT assumes a relativist ontology, highlighting the role of reflexivity, acknowledging the multiple standpoints of both participants and researchers, and situates research within its context.

### Participants

Participants had to self-report PPU, be over 18 years of age, and be English language speakers. There were no exclusion criteria other than the use of extreme or illegal pornography.^1^

The recruitment process involved purposive sampling with snowballing, and as the study developed, theoretical sampling. Theoretical sampling is central to grounded theory and involves the researcher seeking additional participants, and/or amending data collection to illuminate existing categories and in turn the emerging theory (Bryant & Charmaz, 2007).

Recruitment adverts, indicating the methodology (i.e., survey and optional interview), were posted on online rebooting forums and social media sites specific to PPU.

Recruitment was later amended when it became apparent that the sample needed to be more diverse in terms of how participants conceptualized their difficulties. Therefore, general social media, survey sharing sites, and posters (displayed in student areas at London South Bank University) were used to recruit participants. Once permission was granted via email, several pornography sites also posted about the study.

A total of 258 participants completed the survey and 21 were interviewed (see Tables 1 and 2). On the PPUS, participants (*N* = 258) had a mean score of 33.37 (SD = 12.02), min 2, max 60, indicating the sample scored very highly—65% of the sample were classed as help seeking.

**Table 1:** Demographics *N* = 258/*N* = 21

	*N* = 258 (%)	*N* = 21 (%)		*N* = 258 (%)	*N* = 21 (%)
*Age*			*Ethnicity*		
18–24 years	41	43	White	73	90
25–34 years	34	24	Asian	13	5
35–44 years	12	24	Latino	6	0
45–54 years	9	5	Mixed	3	0
55–64 years	3	0	Black	2	0
65–74 years	1	5	Other	2	5
*Gender*			*Employment status*		
Men	90	90	Employed	50	43
Women	8	10	Student	36	24
Transgender male	1	0	Unemployed	9	24
Non-conforming	2	0	Other	5	10
*Sexuality*			*Highest level of education*		
Heterosexual	74	81	Less than high school	3	0
Gay or lesbian	7	5	High school graduate	11	10
Bisexual	16	14	Some college	27	19
Other	3	0	Undergraduate degree	29	48
*Religiosity (are you religious?)*			Postgraduate degree	25	19
Definitely yes	18	14	Doctorate	3	5
Probably yes	11	5	*Living circumstances*		
Might or might not	13	24	Live with romantic partner	19	5
Probably not	15	29	Live with own family	8	14
Definitely not	43	29	Live with friends	14	14
*Relationship status*			Live with family members	33	38
In a serious relationship	39	33	Live on own	26	29
In a casual relationship	7	10	*Country of residence*		
Frequent partners	2	0	UK	36	29
Single	53	57	N. America	24	33
			SE Asia	20	0
			S. America	5	0
			Australasia	5	5
			Others	11	29

**Table 2:** Use of pornography *N* = 258/*N* = 21

	*N* = 258 (%)	*N* = 21 (%)		*N* = 258 (%)	*N* = 21 (%)
*Use of pornography in the last six months*			*Time using pornography per week*		
Several times a day	19	14	11 + hours	5	10
Every day	17	10	6–10 hours	12	19
4–6 days a week	27	24	3–5 hours	22	19
2–3 days a week	15	0	Between 1 and 2 hours	20	10
1 day a week	3	10	Up to 1 hour	17	10
Several times a month	7	19	30 min or less	13	10
At least once a month	3	5	Not applicable	12	24
Every couple of months	8	19	*Times attempted to reduce using pornography*		
*Times attempted to quit using pornography*			More than 5 times	53	62
More than 5 times	49	43	4–5 times	12	5
4–5 times	7	10	2–3 times	16	24
2–3 times	19	33	1 time	5	0
1 time	9	5	Never	13	10
Never	17	10			

All participants (*N* = 258) reported watching pornography online, some also used DVDs/magazines/erotic books/adult movie theaters

All participants (*N* = 258) watched pornography alone, 17 also watched with a partner, and 8 with friends

71% of participants (*N* = 258) masturbated to orgasm whenever they watched pornography, 7% three quarters of the time, 21% half the time, 11% a quarter, 3% reported not masturbating

The journals (N = 258) indicated that pornography was used for a median of 45 min [average time on Pornhub in 2023 was 10 min (Pornhub, 2023)]. The 258 journals contained 24,882 words in total, a mean of 96 words per journal.

Four interviews were in person and the rest were online (data collection occurred during the Covid19 pandemic). The mean duration of the interviews was 65 min (min. 48, max. 82). The interviewed participants (*N* = 21) had a mean PPUS score of 35 (SD = 11.08), min 2, max 55.

### Measures and Procedure

A link or QR code took participants to Qualtrics. After informed consent, participants were asked: “Do you consider yourself to have problematic pornography use?” After confirmation, specific demographics following Kraus et al. (2017) were obtained to contextualize the data. Participants then completed the Pornography History Questionnaire (Rosenberg & Kraus, 2014) and the Problematic Pornography Use Scale (PPUS; Kor et al., 2014).

Using Qualtrics, participants were then asked to complete a journal of pornography use. The purpose of the journal was to allow for functional analysis of the perceived problematic behavior. Functional analysis is an idiographic model of formulating clinical problems, particularly the maintenance of the problem. Participants were asked to give contextual information, then using several free text boxes participants recorded their thoughts, feelings, and behaviors, before, during and after using pornography.

All participants were then invited to participate in a semi-structured interview, based upon a standard cognitive behavioral assessment (Grant et al., 2008)—this approach was used to gather data pertaining to the development of PPU, to formulate their difficulties with pornography. All interviews were conducted by the first author. The interview questions were amended accordingly to help category development. Toward the end of each interview, a preliminary verbal formulation was presented, leading to further discussion and amendment—a form of member checking. All interviews were transcribed, and NVivo was used to store and organize the data.

### Data Analysis

The analysis of data was guided by the constructivist approach to grounded theory. There was simultaneous data collection and analysis. All data were analyzed by the first author, with guidance and critique provided by the second and third authors.

Memos are akin to field notes. Memoing occurred throughout the research process and their use helped both guide and illuminate the analysis with the researcher’s reflections. In addition, the preliminary formulations and feedback were transformed in detailed memos, leading to a triangulation of data sources: interviews, journals, and detailed memos.

Grounded theory coding consists of two phases: initial/focused, and theoretical coding. Line by line coding of the interview data produced several hundred unique codes. The codes were scrutinized, similar codes were reviewed and if appropriate merged together and new focused codes created. “Constant comparative methods” (Glaser & Strauss, 1967) were used throughout the stages of coding. This meant comparing data with data, codes with codes, both within each participant account and between participants, an iterative process.

A pragmatic decision was made to see the journal data in a more realist perspective. The early stages of analyzing the journal data used an inductive method during the first twenty journals. The codes produced were then used deductively as a framework to code the rest, any new codes were then additionally used as a template.

Theoretical coding aims to conceptualize how focused codes relate to each other (Charmaz, 2006), and to develop categories. The focused codes derived from the journal data were amalgamated with the codes from the interview data, with the detailed memos helping to give a structure to this process. The focused codes were developed into categories when there was convergence of ideas. Fully utilizing the concept of theoretical sensitivity, how categories related to each other, the categories were developed and became more explanatory. The process of data collection, coding, and category formation continued until there was theoretical saturation; whereby new data did not expand or create any categories (Charmaz, 2006, 2014). A narrative was then created linking the categories together.

## Results

**Fig. 1:**
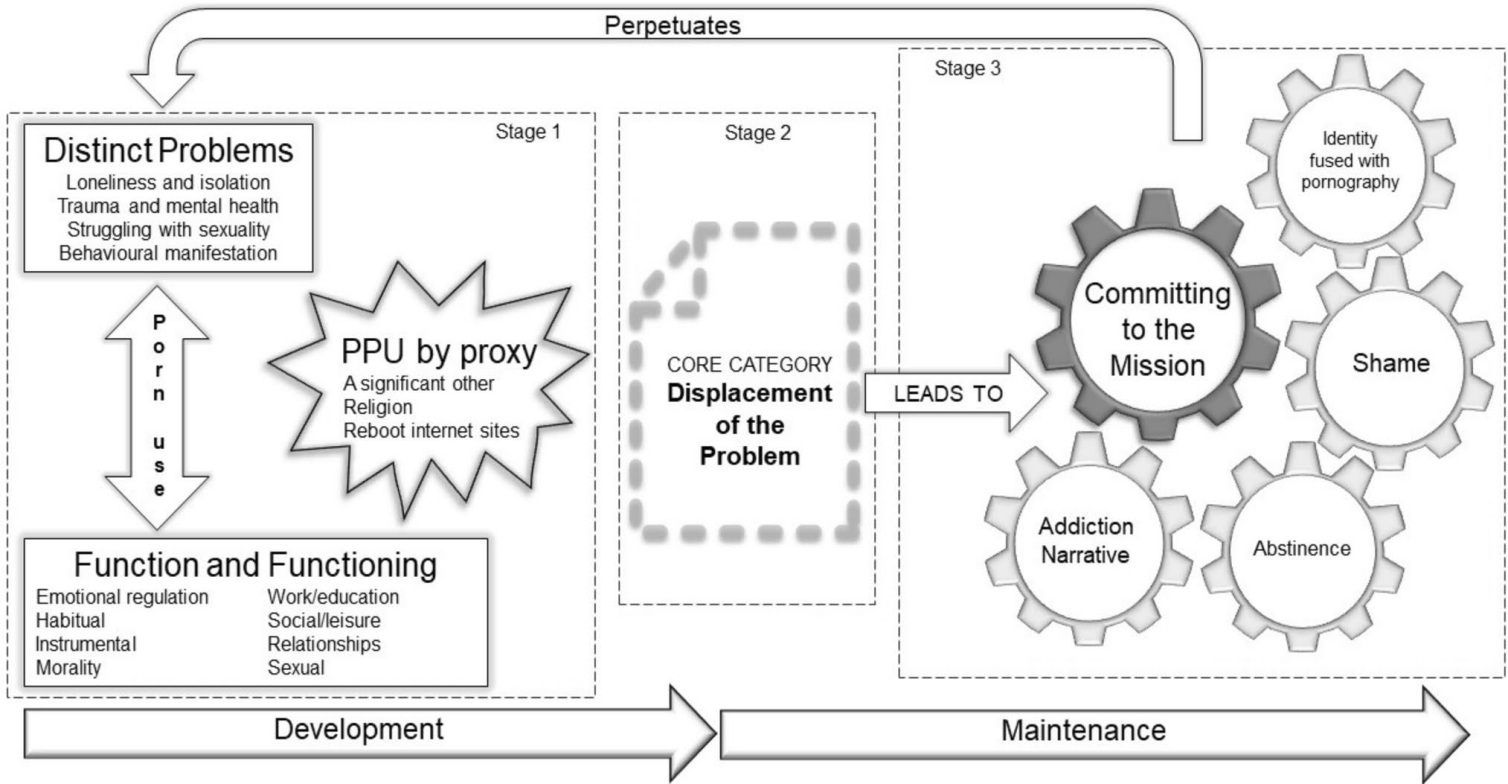
The constructivist grounded theory of problematic pornography use

This constructivist grounded theory of PPU suggests that the participants had preexisting problems before they identified their pornography use as problematic. The pornography use itself may have been causing distress and dysfunction, however alone it did not account for the totality of the participant’s experience. The realization of “having PPU,” or being addicted to pornography, is put forward as occurring due to an external factor. The participants seemed to adopt their morality concerning pornography from “the other,” a relationship or more commonly a reboot forum. Upon this realization the participant’s displaced their preexisting problems. The participants began to see their use of pornography as their main problem and embarked on mission to conquer their addiction. This mission distracted participants from their underlying displaced problem. Most participants were unable to “complete” their mission and felt worse for their failure, perpetuating their feelings associated with their distinct, underlying problems. The theory has been sub-divided into three stages (see Fig. 1):

Each stage is now presented including verbatim statements from participants where appropriate.^2^

### Stage 1 of the Grounded Theory

This stage of the grounded theory explains how the participant’s PPU developed over time. The process begins with an interaction between the category “distinct problems” and the category “function and functioning”; both categories outline the difficulties faced by the participants. The difficulties are labeled by the participants as PPU or pornography addiction, through an external mechanism. This process of identifying with the concept of PPU/addiction constitutes the category “PPU by proxy.”

#### Distinct Problems

Before they considered their pornography use problematic, all interviewed participants reported having problems in their life, or some form of psychological distress or disorder. Four focused codes were constructed:

##### Loneliness and Isolation

Many participants reported feeling lonely, struggling socially, recounting a feeling of being isolated from the world. Several participants adopted the persona of the outsider, struggling with their mood as a result. One commonality was a difficult childhood, feeling different at school, being introverted, with some being bullied. Feeling alone and being too anxious to socialize, participants turned to pornography, replacing the need for connection with others, a safer alternative as compared to the unknown actualities of society.

##### Trauma and Mental Health

Some participants, to a greater or lesser degree, had experiences they saw as traumatic, or they had more severe mental health issues. For one of the participants their trauma influenced their pornography use; they used incest themed pornography as a way of having a meaningful connection, fulfilling a need. Another turned to pornography after a trauma, an attempt to block out painful feelings. One participant had a traumatic childhood and later developed OCD, feeling that there was something wrong with them. This influenced their sexual behaviors, using sex and pornography as a way of gaining a sense of belonging with others. OCD was prominent for another participant who used pornography to combat their intrusive thoughts.

##### Struggling with Sexuality

For a couple of participants, although having their own mental health or social difficulties, their distinct problems seemed to focus upon their struggles with their sexuality. One participant was insecure about their sexuality and body image. Their pornography use became a way of fantasizing about an idealized self. The other used pornography as an outlet for their sexuality. Growing up in the 1970s, they felt they could not express themselves as a bisexual, after marrying a woman, they turned to gay pornography, and later to meeting up with men for sex. They self-identified as a sex addict, rather than resulting from their inhibited sexuality and relationship to sexual activity.

##### Behavioral Manifestation

Some participants had developed certain masturbatory techniques over the years. Rather than the distinct problem being a set of life experiences and associated emotional difficulties, it can be said that their distinct problems were of a behavioral nature.

Erectile dysfunction was seen as influential in the development of PPU. This was particularly the case for two participants. One had a set routine of pornography use, prone masturbation, likely to be causational in his subsequent ED. The other had an operation for his tight foreskin which meant his first sexual experience was painful. This in turn created anxiety which led to ED.

This category of distinct problems proposes that all participants had a preexisting problem prior to identifying with the concept of PPU. Self-reported PPU may only encapsulate a limited set of difficulties. All participants expressed accounts of current and historical distress that by themselves can be seen as problematic. The analysis now turns toward the participants’ pornography use itself; exploring how and why the participants used pornography, what was the purpose, and what were the impacts of using pornography.

#### Function and Functioning

Using primarily the journal data, four focused codes were constructed to demonstrate the various functions of using pornography.

##### Emotional Regulation

Many participants were seen as consciously attempting to change how they felt:

Boredom, depressed feeling, wanting to fill a void with stimulation. (P53).

Wanting to fill a void is a clear example of making a considered decision with the intention of changing affect. Several participants were mindful of how they felt before using pornography:

How alone and sad I feel (P103).

While some were more aware of their thoughts:

I was thinking badly of myself. I'm of little worth: unattractive, unfunny, and generally an unlikable person (P57).

While using pornography the majority enjoyed what they were doing, however most felt negative afterward. Typical quotes included:

Even more depressed than when I started. I felt super fatigued and just empty (P220).

Felt like a total piece of trash for relapsing, disgusted and angry with myself (P22).

Some participants were ‘successful’ in regulating their emotions. The odds of success were approximately 1:9. This may suggest why many participants used pornography multiple times a day—more chances to reduce their painful feelings.

##### Instrumental

These participants also had an intention underpinning their pornography use, seeing pornography as a “means to an end.” Many used pornography as a sleep aid, for example P141 stated:

I knew I didn't want to look at pornography or masturbate, but I knew I needed to in order to fall asleep.

Some participants used pornography as a reward for completing a difficult task:

When I finish what I'm doing, I can take a break and view some porn and masturbate (P197).

A few as a way of punishing, or getting back at their partner, for example P159 recorded:

I was thinking about how I would be justified to act out because I was just rejected by my wife.

All were considered decisions to use pornography. For many, the outcomes were as they predicted, for example, those that used pornography to get to sleep felt tired and fell asleep.

##### Habitual

These participants did not seem to make a considered decision. They used pornography in a habitual manner, as part of a set daily routine. Several participants felt “powerless” regarding their pornography use, as if it was an automatic process. Many felt that they were just going through the motions. P178 was clear about their habitual use:

I had woken up and was on social media. I started masturbating out of habit.

Most participants regretting their habitual use, several did not really get much out of it. P186 described:

A weak orgasm [being] rarely satisfied.

This excerpt is suggestive of a perfunctory process. P60 stated the temporality of the experience:

I just moved on to thinking about work.

Interview P4 really highlighted the automaticity involved:

I come home and then my brain like computer, nobody home equals porn.

The use of a computer analogy, along with the associated speech, adds to the idea of an automatic process.

##### Morality

Many participants had not been feeling particularly negative beforehand but felt guilty afterward.

P138 gave a typical account of the beginning of the process:

I felt horny. I started thinking about how much I'd like to watch something hot.

And P71 gave a typical account of the end of the process:

I felt intensely guilty about looking at porn and I was disgusted I did it.

This was described as a post hoc judgment about what they had done, moral disapproval, summed up nicely by P56:

This is not OK, this is disgusting. I definitely need to go to confession and stop this once and for all.

The interviews highlighted temporal aspects of the participant’s pornography use; an aspect the journals could not. Many participants described how the function of their pornography use had changed over time, from excitement, or expressing their sexuality for example, to becoming a coping mechanism. P2 when describing their teenage use of pornography stated:

I thought it was great, I thought that anybody could look at it any time, there is so much free porn. I thought that it would make me feel good, so why not.

They then described how this changed:

I would have more of an emotional reaction, if I’d had a bad day at school, or if I’d been on assignment or something, I knew I would feel better after I orgasmed.

P11 gave an insightful account of how their pornography use changed over time:

Initially I used it for pleasure. But I was also using it more and more heavily as a thing to help me get to sleep. Then afterwards, it was a thing I used to help me get out of bed in the morning. It got worse to the point where I would use it just to give me a little boost, or just to turn my mind off. I found myself using it as a something to help me get through depression basically.

##### Functioning

The word functioning is used here as it relates to the idea of social indicators of well-being (Andrews & Withey, 1976), the ability to function or perform across different life domains.

When talking about the impact upon their functioning, the time spent using pornography was prominent. Most participants interviewed referred to the duration of their pornography use rather than the frequency. For example, P10 stated:

I'm not proud of this, but my longest session is like thirteen hours or something. Of course, I had breaks, no normal human being could do that for thirteen hours straight.

Using pornography for a long period of time is not necessarily problematic in of itself. However, time spent using pornography came with costs. Many described how they had “wasted time.” They felt that they had prioritized their pornography use over other activities. Some had replaced the need to be in relationships, or their expectations and way of being with others had changed. Some reported how it had ended relationships.

While not diminishing the participants distress and concern, often the direct impact appeared vague. The impact of the pornography use was elusive when seen in combination with the participant’s distinct problems.

#### Problematic Pornography Use by Proxy

This category proposes that all participants labeled themselves as having PPU or being addicted to pornography through an external act, someone else making the decision, or in other words “by proxy.”

Several participants were told they were addicted to pornography by a romantic or sexual partner. A few by different professionals, in one case a psychologist, in two others by sex workers. Religion can be seen as PPU by proxy in a wider sense; for example, P19 saw any masturbation as “*sinful*” and pornography itself as immoral.

About half the participants sought help online after being concerned about their pornography use. Once online, given the search engine algorithms, the participants found themselves on rebooting websites, forums, and subreddits. For many, this was the first time they had heard the phrase “porn addiction.” Participants reported that they found this enlightening, or as P1 stated*:*

Something profound in my life was coming across NoFap. It really made me think about things.

Participants identified with the science sounding information that gave the reboot sites gravitas. P20 discussed how they started to understand how their problems were related to pornography:

I looked at this PornFree subreddit which was cool. I started using all these different resources online like Your Brain on Porn. A lot of it resonated with me, I understood how this rewiring is happening, and this dopamine rush and all this addictive cycles.

P11 was among the clearest in their discovery of a new way of thinking:

Before I found it [PornFree], I didn't even consider it [my pornography use] a problem. Actually, I would say I was blind to it really.

While initially helpful for some, the advice from the online reboot sites was not enough to resolve the participant’s problems.

### Stage 2 of the Grounded Theory

This stage focuses upon the core category of the constructivist grounded theory.

#### Displacement of the Problem

The external process of identifying as a pornography addict seeped into how participants constructed their problems, a re-attribution, away from their distinct problems toward the concept of addiction. All participants displaced their distinct problems to a greater or lesser degree—perceiving their pornography use and/or addiction to be the problem rather than their underlying distinct problems, a redirection from an original source onto another.

Before using pornography regularly, P7 described themselves as follows:

I'm a terrible person. I know for fact that I'm a very selfish person. I'm also an introvert. A lot of the time, I will seclude myself away, just to do whatever on my own.

Through describing these beliefs, P7 indicates the global nature of their self-concept, *a terrible person.* Once identifying as an addict, P7 attributed their problems to their pornography use, rather than acknowledging they had been there for a long time:

I don't have a lot of confidence because of pornography–I'm this freakin weirdo that goes home and jerks himself off.

The original driving cause of the poor functioning becomes less apparent for participants, with all problems being seen through the lens of addiction.

Likewise, P2 had a difficult childhood, being diagnosed with Asperger’s syndrome and depression. They began using pornography as a teenager, seeing themselves as addicted after finding NoFap online. They stated:

My problem with pornography is that it makes me happy not doing anything. I’ve found that if I abuse it, it becomes difficult to think, feels like my head is stuffed with cotton padding and like all of my thoughts are just more sluggish than they should be.

The way in which P2 describes how they feel after using pornography could be called cognitive deficits. Their ability to think clearly is affected, they report a lack of motivation. Both deficits are often associated with a diagnosis of depression. Therefore, there seems to a blurring between causative factors for how they feel. P2 generalizes the impact of pornography, possibly to externalize their problems:

The influx of pornography basically over the last 50 years is unlike anything that has ever happened in human history. I think it’s had a very negative impact on the emotional wellbeing of a whole lot of men.

It seems that P2 may be holding on to something more tangible, that pornography is the cause of their problems. It is perhaps harder for them to look inward and reflect on more internal causative issues.

P13 described themselves as introverted, an outsider. They were bullied at school and lacked confidence, describing themselves as follows:

I would have liked to be a guy who goes out with girls, but I've always been somewhat introverted, and never really been that quick witted.

Looking back on their life P13 regretted their pornography use:

I wonder so much how things would have been different if I had never, ever looked at porn.

Although they had already stated being reclusive before using a lot of pornography, P13 sees their pornography use as the main issue in their life that held them back. They stated:

I don't know where the damage ends, and I begin. I wonder so much how things would have been different if I had never, ever looked at porn

This powerful statement implies that the participants blamed their use of pornography for their lifelong problems. This was a common theme, the idea that ‘if I had not used pornography then…’. For many, their pornography use had got in the way of some kind of self-improvement; however, what or who they would have been without pornography remained vague. It was almost as if pornography had become a justification for not achieving their undefined life goals. These ideas ignore their distinct, underlying issues, which arguably account for their difficulties in life.

### Stage 3 of the Grounded Theory

This stage of the grounded theory begins after the participants had displaced their distinct problems with being addicted to pornography. The distinct problem was no longer their focus, being addicted to pornography was now seen as their main issue.

#### Committing to the Mission

Four focused codes were constructed to explain how participants battled their perceived addiction:

##### Identity Fused with Pornography

Several participants saw pornography as an important aspect of their psyche. P9 was clear in how they understood their relationship with pornography:

Why do I need to do it to the extent and to the degree that I do it? It's part of my identity.

Pornography as something intrinsic to the individual was put forward by P6:

It's hard when it's been internalised for so long to put it where it belongs. It has become a part of who I am. Who would I be without pornography?

This aspect of becoming one with pornography was frightening for P19:

It's just scary to think that it's something that is a part of me forever.

And confusing for P20:

I've been trying to slowly work out what sexual desires are mine and what sexual desires porn is planting in my brain.

P2 made a definite declaration:

My entire life focused around pornography.

If participants had almost fused their identity with pornography, then this indicates that separating from it, or stopping using pornography would be difficult.

##### The Addition Narrative

Many participants related their concept of addiction through a medical lens, that the “disease” of addiction was having an impact upon their biology. P10 summarizes this idea nicely:

The addiction, how it works on your brain, about the brain chemistry, how the dopamine receptors work and all the other stuff, how the deltafosb accumulates in your brain. It has helped at least somewhat in realising why I feel this way.

On the surface, the above excerpt suggests that P10 found learning about pornography addiction useful. However, *this way* implies one way of understanding their experience, a way that may or may not be helpful.

Other biological terms were frequently used by participants. Rather than just using the language, some participants also gave a biomedical account of why they had their difficulties:

Maybe some brains are more susceptible to pornography addiction (P4).

If the narrative was very strong and all-encompassing then this would leave little room for a competing narrative. Once addiction had been viewed within a biomedical framework, several participants started to make analogies to substance use. While others used addiction related ideas:

To get the feeling, or the hit, I suppose that I needed (P18).

It feels like I’m an ex-smoker or ex-drinker where I still get a lot of craving pangs (P11).

I think I hit rock bottom in some way… In a way I was doing it partially for them and not completely for myself. (P10).

P2 felt powerless:

I was realized that it was bad for me, but I wasn’t strong enough to stop myself.

A strong addiction narrative leaves little room for the competing narrative that it was their distinct problems that needed addressing. Once participants took on the addiction narrative there seemed to be a logical leap into a prescribed journey toward abstinence.

##### Abstinence

The association of feeling addicted to pornography and wanting to be abstinent was clearly expressed by many participants. However, the journey toward abstinence was a difficult process:

I was very focused on trying to quit pornography and I couldn't do it. I could take two weeks off or something, but that was the best I could do. (P14).

Requiring much effort:

A huge thing for me, it's been really like admitting that this is like a real thing for me, and I have to put a lot of effort into it. (P20).

P9 expressed their desire to stop, and their disappointment in not being able to so:

I’ve tried to stop plenty of times before, but finally I did it. I told my friends that I was going to do it. I did a tracker on it. Literally the first day I tried to do it I break it. I just kind of sat back and reflected, like what the fuck.

Different strategies are used, a tracker, telling friends, however P6 looked inward to try to understand:

I guess for me being in recovery means having a healthy sexuality. That's something I really struggle with. There's part of me thinking it'd be far easier if I could just remove all of those urges, if everything could be taken away. So I could just be nonsexual, I wouldn't have to deal with any form of sexuality.

In the above excerpt, P6 indicates that they would prefer not to have any sexuality, rather than live with their pornography use. Given the difficulty in being abstinent from what can be seen as an expression of their sexuality, most participants had experience of what they called ‘relapse’ (see Table 2 for the frequency of attempts to quit or reduce pornography). However, the concept of relapse was not consistent. Some found themselves questioning what a relapse is:

If I'm watching Game of Thrones, am I relapsing? (P19).

This excerpt indicates, for at least some participants, that the boundary between abstinence and relapse was blurry. If the relapse is not defined for the individual, then there would almost be a constant threat and likelihood of relapse, thus maintaining the idea of being addicted to pornography.

The longer participants maintained their abstinence the more disappointed they were when they relapsed:

That's why I've been so annoyed with myself, because I had a good streak going that you want to keep going. The problem is you get so much more sad, frustrated and discouraged when you fail. The problem is that you’re more afraid of restarting (P19).

Here, it is suggested that the disappointment arises not only from using pornography but by breaking a commitment not to use. There seemed to be some conflation between wanting to quit pornography as it was problematic and wanting to quit pornography to achieve a goal. P1 is a good example of this process. P1 found it difficult to describe the exact problems with their pornography use, but also described beating the addiction as their number one priority.

Abstinence is a key aspect of reboot sites, as suggested by the names: NoFap, PornFree. The role of reboot communities in the maintenance of the participant’s PPU is complicated. Some found it useful, others did not. However, within this grounded theory, it is proposed that the reboot sites contain an unscientific philosophy and promote an approach which lacks any agreed evidence base. The approach is seen as perpetuating the participants’ concept of addiction, and worsening how participants felt when they relapsed.

Irrespective of whether the participants found the concept of addiction helpful or achieved abstinence, these factors were instrumental in why they considered their pornography use problematic, and why their self-reported PPU persisted. Even though some saw themselves as in recovery they still identified as having PPU, whether it was “active” or not.

##### Shame

The idea of shame as presented here is multifaceted. Shame is firstly thought as “before the self,” then “before the other,” and finally in relation to time.

Some participants, for example P18, felt a general sense of shame, instantly regretting what they had done:

I mean shame, its sometimes pretty filthy and you think what the hell have I just done.

For P9, there was a stronger sense of disgust:

I lay there, and I just feel dirty, gross, heavy. It's almost like you want to go and try to do something to make up for what you just did.

Despite the immediate feelings of shame some participants used pornography again, perhaps to avoid the painful shameful feeling itself:

I would finish bingeing porn and I feel so guilty about it and shameful and be like, I'm never going to do it again. And then I would like, sometimes that day, do it again (P20).

For P20, his sense of shame was in relation to how he saw himself, and how using pornography contradicted this:

I just feel like a massive hypocrite in a lot of ways. Because watching porn, especially the type of porn that I would watch, does not align with my values and how I am in any other part of my life whatsoever.

For some participants, their sense of shame originated from the other. As outlined in the category ‘PPU by proxy,’ this was an external process, originating from a grand morality such as religion:

I’ve always attributed it [pornography use] with a bad feeling. I knew it was kind of wrong, been a lot of guilt. Part of it is religious, because it's against religion to masturbate. (P19).

For P13, using pornography was a ‘dirty secret.’ They reported how their secret influenced how they were around other people and how this made them feel:

Using pornography would make me really nervous around girls because I would feel like I'm having all these dirty thoughts and I'm scared that they can tell somehow, they could tell it's icky and creepy.

Here, P13 feels their internalized shame concerning their use of pornography can be picked up by other people. This is such an uncomfortable feeling that it impacts upon them socially.

Rather than an internal or external aspect to their shame, some participants expressed their shame as regret over a loss of time and opportunity. P5 stated:

I hate thinking about it at times, how much time I've wasted. I hate to think how much time and money I've wasted in my life, it's just too horrendous to think about sometimes.

Being ashamed, having regret or disappointment was a common theme among the participants. Rather than a sense of disgust or shame before another, a relapse could be conceptualized as regret for failing more generally. Several participants felt a sense of shame at not being able to stop pornography. They were disappointed in not achieving this task, failing at achieving abstinence.

As all participants had some form of distinct problem unrelated to their pornography use then an increase in shame, guilt, a sense of being a failure made them feel worse, exacerbating the distinct problem.

## Discussion

The originality of this grounded theory consists of reformulating the concept of PPU into an alternative explanation of distress and dysfunction. However, there are elements that relate to existing research. The dual systems model (Strack & Deutsch, 2004)—addictive behavior is governed by the interplay of two systems, a reflective system (conscious decision-making), and an automatic system (responding to habits and impulses), relates to the category ‘function and functioning’ outlined within this grounded theory; how the participant’s use of pornography varied, and changed over time for the participants. Likewise, Grubbs et al. (2018) found that rather than the amount of pornography used, individuals saw themselves as more addicted to pornography if they had strong beliefs against using it, including religious beliefs (Grubbs et al., 2015); therefore, using pornography would be a moral transgression. This was a theme across different categories.

The category “PPU by proxy” relates to thinking from a media studies perspective. Room (2014) describes how in modern culture “addiction” gives an explanation of the inexplicable. It is also seen as “alien” to the “real” character, allowing the audience to sympathize. Within this grounded theory, participants found it easier to adopt the concept of addiction, rather than focusing upon their distinct problems.

The word “displacement” was used to describe the process occurring within the core category. In psychoanalytic theory, displacement is a defense mechanism, first introduced by Freud (1894/1964). Displacement involves the unconscious redirection of an emotion or object from its original target to a substitute target that is less threatening or more acceptable (Bowins, 2004). Dodes (2009) sees addiction as a form of displacement, the addictive behavior being the substitute action. This process can be called a narcissistic defense (Kohut, 1971), it protects the ego. This grounded theory conceptualized the “displacement of the problem” as a process of re-focusing of attention, rather than as a narcissistic defense. Psychoanalytical theory pertains to the movement of emotions, whereas the core category suggests the movement or re-attribution of “problems.” By this, it is meant that within this grounded theory, there is change of what the problem is, not just emotions.

Identity as an addict was constructed as an important aspect of this grounded theory. Social identity theory (Tajfel & Turner, 1979) elucidates how this aspect of the participants’ identity was developed. Many participants took on the addiction narrative when finding reboot sites. Within the reboot communities PPU is seen as an addiction, competing narratives are rarely present. Folk concepts concerning addiction (Morris, 2022) were expressed by many participants. If spending time within the reboot communities, then the participants’ identity as an addict would be strengthened, and as proposed in this grounded theory one of the ways in which PPU is maintained. Being an addict involves addictive behaviors, likewise, being in recovery includes abstinence (Buckingham et al., 2013). Being part of a recovery group (such as rebooting) leads to persisting with abstinence despite relapse (Best et al., 2014). The discussion of abstinence within this grounded theory is not intended to suggest that abstinence within addiction is unhelpful, but within PPU it is seen as maintaining factor, further distracting participants from their distinct problems.

The grounded theory highlights the importance of taking a formulation-based approach, and not assuming that a traditional addiction framework is applicable to the issue of PPU. It is suggested that the phrase “pornography addiction” maybe unhelpful. The theory prioritizes the underlying issues, classed as the “distinct problems,” as one of main factors for intervention. Beliefs concerning pornography, and responses to relapse are also seen as important areas to explore within the therapeutic context.

As a new theory to the research literature on the subject of PPU, there are opportunities for further research. The theory as it stands cannot be generalized, practitioners could find it a useful aid to therapeutic work, but more research needs to be conducted. Further qualitative research focusing upon the acceptability of the theory to those self-reporting PPU would seem intuitive.

While this study has application, there are limitations that should be acknowledged. In terms of the sampling, there were participants who discussed their past use of pornography, they identified as being in recovery from PPU. Several accounts will have been filtered through ideas from the present. However, those in recovery still thought of themselves as “having PPU”; therefore, their experiences were valid in helping to answer the research question.

The data collection timeframe coincided with the onset of the COVID-19 pandemic. Pornhub reported that there had been an increase in pornography use of 11.6% during the pandemic (Pornhub, 2020). Fotinos et al. (2024) suggested that this increase in pornography consumption was due to the inability to engage in partnered sex due to the lockdown policies. As most participants were not in a sexual relationship at the time of interview, then the impact can be assumed to be minimal.

It would have been more methodically rigorous to have more than one person coding the data. However, the research was conducted as part of a Ph.D., having multiple coders was therefore not possible. Ideas and processes were discussed however within the supervisory team, as were issues of reflexivity.

### Conclusion

This constructivist grounded theory suggests for those self-reporting PPU, pornography use is a coping mechanism for underlying problems; the coping mechanism becomes erroneously seen as the problem after engaging with external sources. A process of displacement occurs, resulting in the underlying problems being unattended. Efforts at abstinence increase shame and exacerbate the underlying problems.

The above original statement was created through a rigorous research process using two sources of data generation, including a “clinical” sample, and an inductive approach. The study aimed to create an understanding of the development and maintenance of PPU. Both these aspects are crucial in developing personalized, idiographic, psychological formulations. The theory presented here may help practitioners take a more nuanced approach to their therapeutic work, rather than implementing more traditional addiction-based interventions.

## Data Availability

Upon request.

## Footnotes

1 As defined by U.K. legislation.

2 Vaguely defined words such as “few,” “some,” “several,” or “many” are used to give an indication of regularities and idiosyncrasies in the data, but not an indication of the generalisability of the findings beyond the sample studied (Neale et al., 2014).
